# Tau in the pancreas: understanding the link between type 2 diabetes mellitus and Alzheimer’s disease

**DOI:** 10.1038/s41392-023-01701-3

**Published:** 2023-12-06

**Authors:** Wenlu Li, Steffen Tiedt, Eng H. Lo

**Affiliations:** 1 Neuroprotection Research Laboratories, Departments of Radiology and Neurology, Massachusetts General Hospital, Harvard Medical School, Charlestown, MA 02129 USA; 2 Consortium International pour la Recherche Circadienne sur l’AVC (CIRCA), Boston, USA; 3 Institute for Stroke and Dementia Research, LMU University Hospital, LMU Munich, Munich, Germany

**Keywords:** Endocrine system and metabolic diseases, Neurology

A recent study published in *Molecular Psychiatry* proposes a cellular mechanism that may contribute to decreased insulin production in individuals with type 2 diabetes mellitus (T2DM).^1^ Using a combination of cell culture experiments, animal models and studies in T2DM patients, the authors show that an up-regulation of tau in β-islet cells can inhibit pancreatic insulin secretion through changes in microtubule assembly.^1^

Tau is expressed not only in the brain. For example, it has been proposed that tau in other organs such as lung endothelium may interact with and contribute to dysfunction in the central nervous system.^2^ Salvatore et al. examined pancreatic tissue from control subjects and patients with T2DM, and confirmed the presence of tau in β-cells, where it co-localized with insulin in both control subjects and T2DM patients. Furthermore, pancreatic tau levels significantly increased in T2DM patients and the T2DM mouse model (*db*/*db* mice), highlighting its relevance in the endocrine pancreas in the context of diabetes.^1^ Indeed, insulin secretion, as indicated by two glucose tolerance tests (intraperitoneal and intravenous), was increased in mid-aged (12-month) tau knockout mice. Moreover, specifically knocking down tau in the β-islet induced elevated insulin secretion, indicating the importance of pancreatic tau on metabolic traits.^1^ Of note, pancreatic tau is significantly up-regulated not only in T2DM but also in neurodegenerative diseases like Alzheimer’s disease (AD), dementia with Lewy bodies, Parkinson’s disease, and incidental Lewy body disease.^3^ This raises intriguing questions. Is there a common denominator causing tau to be dysfunctional in the whole body? could pancreatic tau impact brain function through insulin-related mechanisms? Or is it the accumulated tau within the brain in the presence of a neurodegenerative process that triggers the up-regulation of tau in the pancreas (Fig. 1)?

**Fig. 1:**
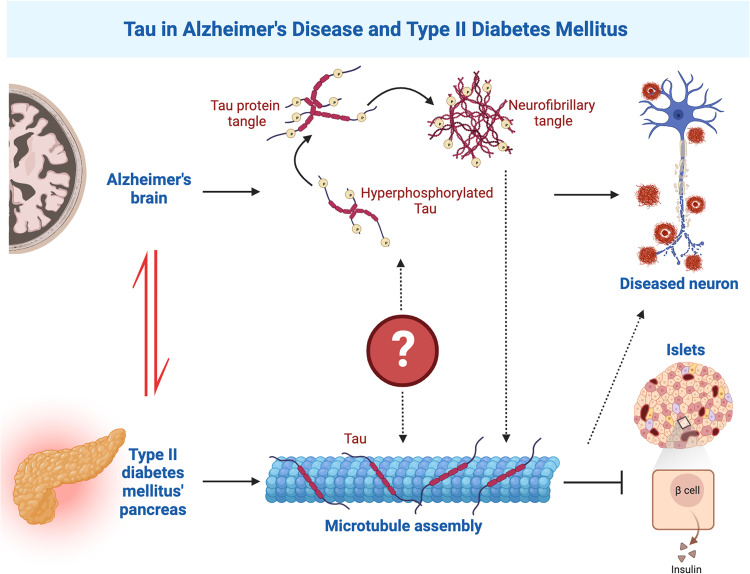
Schematic diagram of Tau in AD and T2DM. In AD, Tau hyperphosphorylation results in neuronal cell death and degeneration. In this study, Tau protein inhibits insulin release in islet cells by promoting microtubule assembly, providing a potential new insight into the molecular links between T2DM and AD

How might pancreatic tau regulate insulin secretion? Tau is a microtubule-associated protein. The authors found a significant reduction in the co-localization of β-tubulin and insulin in the pancreas when tau was absent in islets, with a sparse microtubule network in islet cells. This suggests that microtubule assembly might play a crucial role in tau-mediated pancreatic insulin secretion (Fig. 1). Indeed, colchicine or nocodazole, which inhibits microtubule assembly by binding to tubulin and disrupting the dynamic assembly of microtubules, reduced the peak of blood glucose in a glucose tolerance test (oral) in healthy mice and glucose-treated β-cell-line INS-1.^1^ To investigate the potential of normalizing elevated plasma glucose levels in diabetic models through microtubule disassembly, colchicine was administered to both *db/db* mice and New Zealand Obese diabetic mice. This treatment induced pancreatic tubulin disassembly in *db*/*db* mice, significantly reducing blood glucose levels in a glucose tolerance test (intraperitoneal), accompanied by an increase in baseline insulin levels.^1^

To further investigate whether tau-mediated insulin release might be relevant to human patients, the authors focused on individuals with early-onset Alzheimer’s disease (EOAD) and healthy controls. While serum tau levels (but not phosphorylated tau) and blood glucose levels were positively correlating in healthy controls, no such association was evident in EOAD patients.^1^ While further extensive and controlled cohort studies using more elaborate statistical methods including interaction analysis may be necessary to draw a definitive conclusion, these data are supportive of the potential role of tau in regulating glucose levels and impairment of that mechanism in AD patients.

Notably, another study found elevated insulin levels in the brains of AD patients, particularly in the hippocampus.^4^ Immunofluorescence studies conducted on hippocampal slices from patients at various stages of AD revealed significantly higher insulin levels in neurons that were positive for AT180, an antibody recognizing hyperphosphorylated tau at T231, especially in those with neurofibrillary tangle formation.^4^ Indeed, intraneuronal accumulation of insulin is directly associated with tau hyperphosphorylation and follows the progression of tauopathy.^4^ Whether microtubule is related to hyperphosphorylated tau-mediated neuronal insulin accumulation, or if different mechanisms are involved in brain diseases compared to peripheral diseases warrants further investigation.

How pancreatic tau affects insulin secretion at the molecular level is not yet fully understood. It is important to note that glucose metabolism, insulin sensitivity, and insulin secretion exhibit circadian variation. The central circadian clock regulates functions such as food intake, energy expenditure and whole-body insulin sensitivity. These processes are further adjusted by local peripheral clocks. Specifically, the peripheral clocks within the pancreas play a role in insulin secretion. In addition to the rhythmic transcription of genes controlling insulin secretion, tubulin polymerization promoting protein gene (*TPPP*) null mice exhibited modified profiles of circadian rhythm-related gene expression, decreased melatonin levels, a phase-shifted sleep/activity cycle, and reduced amplitude in activity rhythms compared to wild type mice.^5^ These findings suggest that microtubule dynamics are key to exhibiting proper circadian function. Therefore, it is possible that tau-mediated insulin secretion by the β-cells, through the regulation of microtubule assembly, is intricately connected to circadian rhythmicity. Whether these mechanisms may underlie connections between circadian disruption, diabetes and tauopathies warrants further investigation.

Considering the critical role of insulin in maintaining glucose homeostasis and regulating metabolic processes, coupled with its intricate association with synaptic plasticity, learning and memory, the studies of Salvatore et al. have defined a new pathophysiological pathway that may pave the way for new approaches in comprehending the connection between insulin signaling and tau, and for the development of innovative therapeutic approaches simultaneously targeting diabetes mellitus and tauopathies.

## References

[1] Mangiafico, S. P. et al. Tau suppresses microtubule-regulated pancreatic insulin secretion. *Mol Psychiatry*10.1038/s41380-023-02267-w (2023). 10.1038/s41380-023-02267-w 37735502

[2] Balczon, R. et al. Lung endothelium, tau, and amyloids in health and disease. *Physiol. Rev*. 10.1152/physrev.00006.2023 (2023). 10.1152/physrev.00006.2023 PMC11281824 37561137

[3] Martinez-Valbuena I (2021). Mixed pathologies in pancreatic beta cells from subjects with neurodegenerative diseases and their interaction with prion protein. Acta Neuropathol. Commun..

[4] Rodriguez-Rodriguez P (2017). Tau hyperphosphorylation induces oligomeric insulin accumulation and insulin resistance in neurons. Brain.

[5] Barbato E, Darrah R, Kelley TJ (2021). Tubulin polymerization promoting protein affects the circadian timing system in C57Bl/6 mice. J. Circadian Rhythms.

